# Ventilatory strategy during liver transplantation: implications for near-infrared spectroscopy-determined frontal lobe oxygenation

**DOI:** 10.3389/fphys.2014.00321

**Published:** 2014-08-25

**Authors:** Henrik Sørensen, Hilary P. Grocott, Mads Niemann, Allan Rasmussen, Jens G. Hillingsø, Hans J. Frederiksen, Niels H. Secher

**Affiliations:** ^1^Department of Anesthesia, Rigshospitalet, University of CopenhagenCopenhagen, Denmark; ^2^Department of Anesthesia and Perioperative Medicine, St. Boniface Hospital, University of ManitobaWinnipeg, MB, Canada; ^3^Department of Surgery and Transplantation, Rigshospitalet, University of CopenhagenCopenhagen, Denmark

**Keywords:** cerebral oxygenation, cerebral oximetry, end-tidal carbon dioxide, liver transplantation, monitoring, ventilation

## Abstract

**Background:** As measured by near infrared spectroscopy (NIRS), cerebral oxygenation (S_c_O_2_) may be reduced by hyperventilation in the anhepatic phase of liver transplantation surgery (LTx). Conversely, the brain may be subjected to hyperperfusion during reperfusion of the grafted liver. We investigated the relationship between S_c_O_2_ and end-tidal CO_2_ tension (EtCO_2_) during the various phases of LTx.

**Methods:** In this retrospective study, 49 patients undergoing LTx were studied. Forehead S_c_O_2_, EtCO_2_, minute ventilation (VE), and hemodynamic variables were recorded from the beginning of surgery through to the anhepatic and reperfusion phases during LTx.

**Results:** In the anhepatic phase, S_c_O_2_ was reduced by 4.3% (95% confidence interval: 2.5–6.0%; *P* < 0.0001), EtCO_2_ by 0.3 kPa (0.2–0.4 kPa; *P* < 0.0001), and VE by 0.4 L/min (0.1–0.7 L/min; *P* = 0.0018). Conversely, during reperfusion of the donated liver, S_c_O_2_ increased by 5.5% (3.8–7.3%), EtCO_2_ by 0.7 kPa (0.5–0.8 kPa), and VE by 0.6 L/min (0.3–0.9 L/min; all *P* < 0.0001). Changes in S_c_O_2_ were correlated to those in EtCO_2_ (Pearson *r* = 0.74; *P* < 0.0001).

**Conclusion:** During LTx, changes in S_c_O_2_ are closely correlated to those of EtCO_2_. Thus, this retrospective analysis suggests that attention to maintain a targeted EtCO_2_ would result in a more stable S_c_O_2_ during the operation.

## Introduction

Autoregulation ensures that cerebral blood flow (CBF) is sufficient to meet the metabolic requirements of the brain, but may be challenged by a low arterial pressure, hypoxia and/or hypocapnia (Kety and Schmidt, 1948; Lassen, 1959). Maintaining mean arterial pressure (MAP) within the cerebral autoregulatory range during surgery has been suggested to result in improved patient outcome (Ono et al., 2013). An evolving strategy for control of the circulation during surgery is to maintain cerebral oxygenation (S_c_O_2_), a real-time surrogate for CBF measured using near infrared spectroscopy (NIRS). S_c_O_2_ not only has the ability to identify whether patients demonstrate intact cerebral autoregulation, but also determines its lower limit threshold (Nissen et al., 2009).

Impaired cerebral autoregulation (Larsen et al., 1995), cerebral hyperemia, and increased intracranial pressure (Aggarwal et al., 1994) are all associated with end-stage liver disease and may predispose to either ischemic or hyperemic cerebral injury. Cerebral perfusion and thereby S_c_O_2_, is challenged by the hemodynamic events that can occur during liver transplantation (LTx) (Adams et al., 1987; Larsen et al., 1999; Pere et al., 2000; Van Mook et al., 2005; Nissen et al., 2010; Zheng et al., 2012). In the hepatic dissection phase, there is a risk for hemorrhage. In the anhepatic phase inadequate venous return to the heart and a low arterial carbon dioxide tension (P_a_CO_2_) can occur. This contrasts with the reperfusion phase where increases in P_a_CO_2_ may occur (Pere et al., 2000; Panzera et al., 2006). With clamping of the inferior vena cava (IVC), cardiac output (CO) is reduced by as much as 50%, and this can result in compromised perfusion to vital organs including the brain (Pere et al., 2000). Thus, to facilitate hemodynamic stability and to optimize organ perfusion, veno-venous bypass may be utilized (Shaw et al., 1985). Alternatively, venous return to the heart may be assisted by only partially clamping the IVC (so-called piggyback technique) (Panzera et al., 2006). However, even with the piggyback technique, S_c_O_2_ is likely to decrease by about 15% (Panzera et al., 2006) increasing the risk of cerebral ischemia (Al-Rawi and Kirkpatrick, 2006).

In the anhepatic phase of LTx, the systemic metabolic rate is reduced by ~30% and there is therefore a reduced need for minute ventilation (VE) in order to preserve CBF and S_c_O_2_. Conversely, with reperfusion of the grafted liver, metabolism is restored and the brain may be subjected to hyperperfusion due to enhanced CO_2_ and/or liberation of vasodilating substances (Ejlersen et al., 1994; Skak et al., 1997) that could lead to brain edema, hemorrhage and even death (Van Mook et al., 2005). S_c_O_2_ follows changes in CBF with hyper- and hypo-capnia (Rasmussen et al., 2007) and therefore to maintain S_c_O_2_ during the operation potentially minimizes incidence of post-operative neurological complications (Madsen and Secher, 2000; Pere et al., 2000; Zheng et al., 2012).

In this retrospective observational study, we reviewed S_c_O_2_, end-tidal CO_2_ tension (EtCO_2_), and VE for LTx patients and hypothesized that S_c_O_2_ would decrease in the anhepatic phase of the operation and increase again with reperfusion of the grafted liver. We considered that the data would provide an indication as to what extent VE should be adjusted to maintain S_c_O_2_ and potentially contribute to brain protection during LTx.

## Materials and methods

Data were collected retrospectively for patients undergoing LTx at Rigshospitalet (Copenhagen) from 1997 to 2001. The study was performed in accordance with guidelines provided by The National Committee on Health Research and approved by the Local Ethical Committee (H-2-2014-FSP27) who waived the need for patient consent.

The liver transplantation technique involved clamping of the IVC with lower body venous return supported by a veno-venous bypass from the left femoral vein to one or two arm veins (Rasmussen et al., 1994). Reperfusion of the grafted liver was established by opening the IVC above the hepatic vein, followed by the IVC below the hepatic vein, and lastly the hepatic artery. Reported hemodynamic variables include heart rate (HR) and femoral MAP measured via an arterial catheter (Becton Dickinson and Company, New Jersey, NY, USA) cardiac output (CO) by thermodilution (7.5F; Baxter, Uden, Holland), thoracic electrical impedance index (THI) (*n* = 30) (TI; Caspersen and Nielsen, Copenhagen, Denmark) as an indication of the central blood volume (Cai et al., 2000), and S_c_O_2_ (Invos 3100 Cerebral Oximeter, Somanetics, Troy, MI, USA) along with VE and EtCO_2_. P_a_CO_2_ was not continuously monitored, however, it was assumed that EtCO_2_ reflects changes in P_a_CO_2_ as expressed by the ratio between CO_2_ and the alveolar ventilation. All values were noted every 10 min as recorded in the anesthetic chart. Hematocrit was monitored (ABL 700 Radiometer, Copenhagen) and any administration of packed red blood cells and plasma was performed through a rapid infusion system (Haemonetics, Braintree, MA, USA) to maintain a hematocrit of 30%.

Data from the last 60 min of the dissection phase, first and last 30 min of the anhepatic phase, and the first 40 min of the reperfusion phase of the operation were included in the analysis. Hemodynamic changes from dissection to early anhepatic phase were calculated as the difference between an average over 60 min in the dissection phase and 30 min in the early anhepatic phase. Changes from late anhepatic to reperfusion phase were identified as the difference in average from the last 30 min of the anhepatic phase, and the first 20 min of the reperfusion phase.

**Figure 1:**
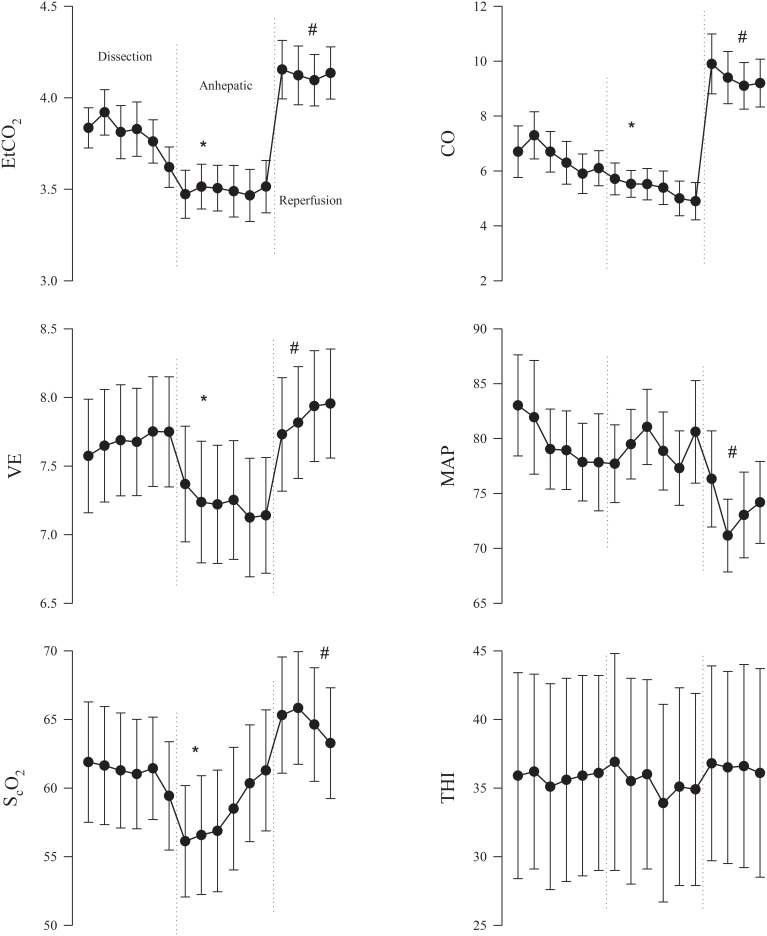
**Mean (±95% confidence interval) for 10th min in the dissection, anhepatic and reperfusion phases of liver transplantation surgery for end-tidal CO_2_ tension (EtCO_2_); ventilation (VE); near infrared spectroscopy-determined frontal lobe oxygenation (S_c_O_2_); cardiac output (CO); mean arterial pressure (MAP); and thoracic electrical impedance index (THI)**. CO and THI reported as geometric means. ^*^P < 0.05 compared to the dissection phase of the operation. ^#^*P* < 0.05 compared to the anhepatic phase.

Distribution of data including variance and probability plots were assessed independently for each patient and the whole population using *Proc Univariate* in SAS 9.2 (SAS Institute, Cary NC, USA). All variables exhibited normal distribution, however, CO and THI were skewed to the right. Thus, we performed a logarithmic transformation (log_10_) on CO and THI-data and relative changes are reported as log(x) − log(y) = log(x/y) (Bland and Altman, 1996a). In Figure 1, CO and THI are presented as geometric means ±95% confidence interval (Bland and Altman, 1996b). We applied an analysis of variance followed by a Tukey–Kramer *post-hoc* test to evaluate changes between conditions and a *P*-value < 0.05 was considered as statistically significant. Association between S_c_O_2_, VE, and EtCO_2_ was evaluated by Pearson's correlation. Since S_c_O_2_ has been reported to decrease with increasing plasma bilirubin (Madsen et al., 2000; Song et al., 2011) that relation was also evaluated with Spearman rank order correlation.

## Results

Forty nine patients, [21 women, 28 men, 53±10 (mean ± SD) years] were admitted for LTx. Twenty six patients had cirrhosis, 5 primary biliary cirrhosis, 4 primary sclerosing cholangitis, 3 acute liver failure, 3 hepatocellular carcinoma, and the remaining 8 patients had other liver diseases. The duration of surgery was 368 min (range; 240–675), representing 141 min (60–465) for the dissection phase of the operation, 83 min (50–250) for the anhepatic phase, and 145 min (70–230) for completion of the operation.

### Anhepatic phase

From the initial dissection to the anhepatic phase of the operation, S_c_O_2_ and EtCO_2_ decreased by 4.3% [(95% confidence intervals: 2.5–6.0%) and by 0.3 kPa (0.2–0.4 kPa; both *P* < 0.0001)] as VE was reduced by 0.4 L/min (0.1–0.7 L/min; *P* = 0.0018). HR, MAP, and THI remained stable (Figure 1). CO was reduced by 15% (6–24%; *P* = 0.0003).

**Figure 2:**
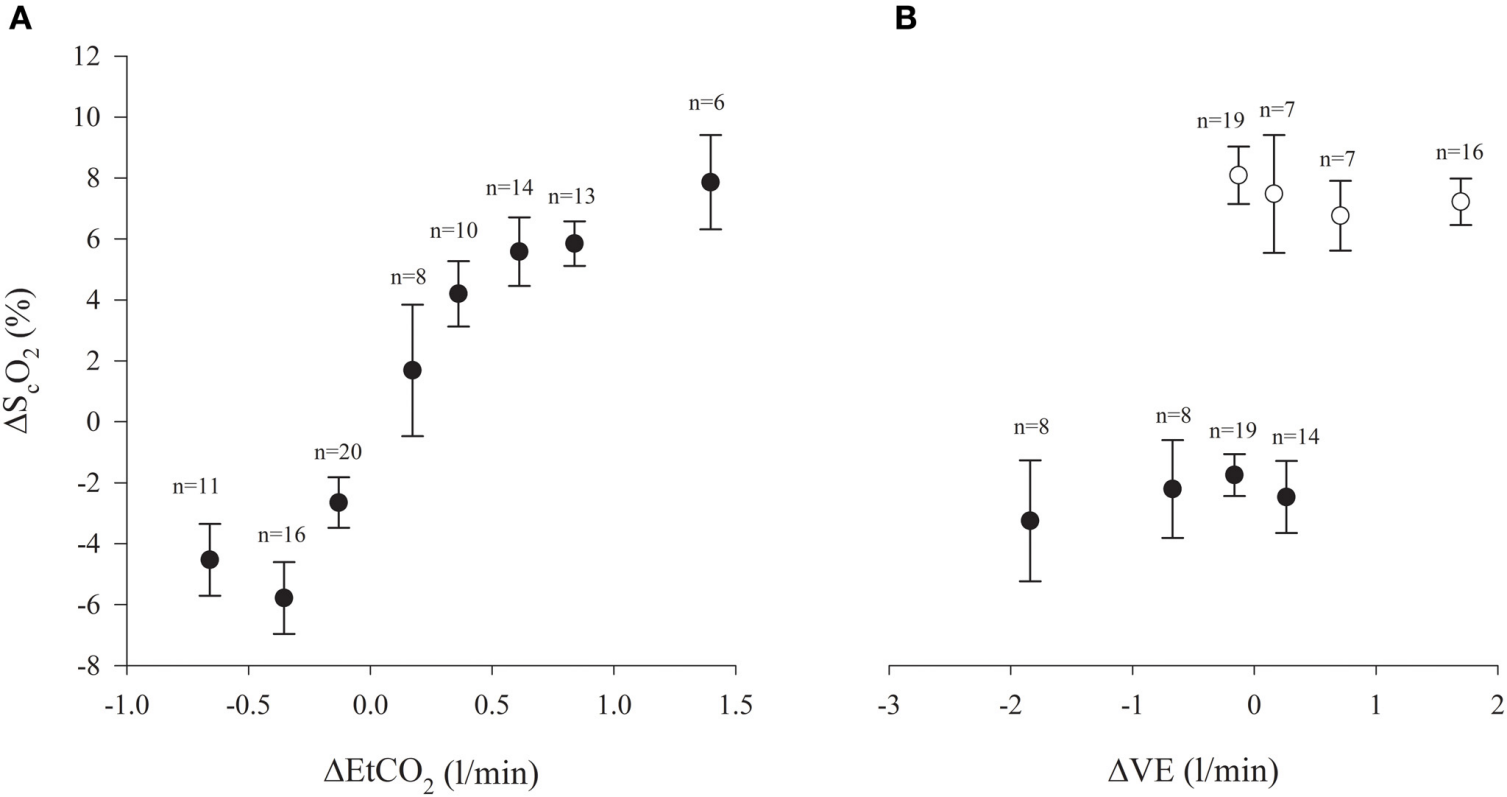
**(A)** Frontal lobe oxygenation (S_c_O_2_) and end-tidal CO2 tension (EtCO_2_) in the anhepatic and reperfusion phases of liver transplantation surgery (% changes from baseline; ± s.e.m.) (Pearson *r* = 0.74; *P* < 0.0001). Number of subjects indicated. **(B)** Changes from baseline (± s.e.m.) for S_c_O_2_ and ventilation (VE). Black symbols: anhepatic phase (Pearson *r* = 0.06; *P* = 0.7). Open symbols: reperfusion phase (Pearson *r* = −0.18; *P* = 0.21). Number of subjects is indicated.

Changes in S_c_O_2_ was correlated to those in EtCO_2_ (Pearson *r* = 0.74; *P* < 0.0001), however, no correlation between S_c_O_2_ and VE was observed (Pearson *r* = 0.06; *P* = 0.7) (Figure 2). In 11 patients, S_c_O_2_ was reduced by more than 15%. We observed an inverse relationship between S_c_O_2_ with plasma bilirubin (Spearman *r* = −0.49; *P* = 0.008) ranging from 9 to 565 μmol/L (*n* = 28).

### Reperfusion phase

During reperfusion of the grafted liver, S_c_O_2_ and EtCO_2_ increased 5.5% (3.8–7.3%) and 0.7 kPa (0.5–0.8 kPa; *P* < 0.0001) as VE was increased by 0.6 L/min (−0.5–3.1 L/min; all *P* < 0.0001) (Figure 1). No changes were observed in HR and THI, but CO increased by 90% (71–110%; *P* < 0.0001). Conversely, MAP decreased by 5 mmHg (1–9 mmHg; *P* = 0.007). No significant correlation between S_c_O_2_ and VE was identify (Pearson *r* = −0.18; *P* = 0.21) (Figure 2), but 13 patients S_c_O_2_ increased >15% compared to the late anhepatic phase.

## Discussion

In this retrospective study of measurements during LTx in 49 patients, cerebral oxygenation (S_c_O_2_), as determined by NIRS, was shown to decrease in the anhepatic phase of the operation and to increase during reperfusion of the grafted liver. Changes in S_c_O_2_ were directly related to the end-tidal CO_2_ tension. Therefore, a ventilatory strategy directed to a maintain EtCO_2_ could ensure stability of S_c_O_2_ during the operation and may, at least potentially, minimize the incidence of post-operative seizures, confusion, and stroke (Madsen and Secher, 2000; Pere et al., 2000; Zheng et al., 2012). Despite bilirubin absorption of infrared light resulting in a low S_c_O_2_, NIRS detected changes in cerebral oxygenation even in patients who were significantly jaundiced (Madsen et al., 2000).

Patients with liver disease are susceptible to alterations in MAP that can frequently result in pressure below the limits of cerebral autoregulation and then may lead to cerebral ischemia. Cerebral oxygenation might further be aggravated by increases in intracranial pressure that reduce cerebral perfusion pressure according to the Monro–Kellie doctrine (Larsen and Wendon, 2008). Thus, it seems to be an advantage if handling of the circulation during LTx involves continuous monitoring of the brain circulation to reduce adverse neurological outcome. NIRS represents a real-time, though indirect, monitor of CBF and indicates its autoregulatory capacity (Nissen et al., 2009; Zheng et al., 2012). In this cohort of LTx patients, S_c_O_2_ was reduced by 4.3% (2.5–6.0%) with IVC clamping (Figure 1), which is likely induced by hyperventilation as indicated by a reduction in EtCO_2_ by 0.3 kPa, albeit VE was diminished by 0.4 l/min. Thus, with the central blood volume maintained as indicated by THI (Cai et al., 2000), a ventilatory strategy guided by EtCO_2_ may avoid cerebral ischemia in the anhepatic phase (Pott et al., 1995), e.g., by keeping EtCO_2_ between 4.7 and 6.0 kPa, arterial CO_2_, CBF, and S_c_O_2_ were maintained (Pott et al., 1995; Zheng et al., 2012). In contrast, no ventilatory adjustment in the anhepathic phase of the operation has been reported to lead to pronounced reductions in P_a_CO_2_, and yet maintained CBF as indicated by transcranial Doppler (Pere et al., 2000). In that study (Pere et al., 2000), cardiac preload was not supported by a venous-venous bypass, while we registered an 15% reduction in CO when the shunt was established. Although we cannot rule out that this reduction in CO may affect CBF and S_c_O_2_, we find it more likely that changes in S_c_O_2_ relate to alterations in EtCO_2_ than to the reduction in CO with the hierarchy of blood flow in the anhepatic phase (Figure 2) (Rhee et al., 2012; Ono et al., 2013; Mahal et al., 2014). In 22% of the patients, S_c_O_2_ was reduced by >15% (relative to the value in the dissection phase) thus lowering the threshold for cerebral ischemia (Al-Rawi and Kirkpatrick, 2006). Similar significant cerebral deoxygenation is reported in up to 50% of patients undergoing LTx (Plachky et al., 2004), and also seen with the use of the piggy-back technique (Panzera et al., 2006).

Postoperative biomarkers of brain damage include neuron-specific enolase and S-100β and they may increase three-fold in patients who demonstrate cerebral deoxygenation (Plachky et al., 2004). S-100β levels are high in patients who develope post-operative cognitive dysfunction (POCD) (Linstedt et al., 2002) and cerebral deoxygenation (>15% relative to baseline) is related to POCD and longer hospital stay (Casati et al., 2005; Ballard et al., 2012; Colak et al., 2014). Moreover, inherent to prolonged cerebral deoxygenation, confusion, somnolence and transient hemiparesis manifest post-operatively (Madsen and Secher, 2000) or permanent neurological damage develops (Philips et al., 1998). Also in patients with acute liver failure, cerebral infarction after LTx can led to long-term hospital care, however, perioperative cerebral oxygenation was not reported for that patient (Pere et al., 2000). In general, patients with encephalopathy have been reported with a 15% higher S_c_O_2_ (Panzera et al., 2006), may be as a result of cerebral hyperemia because of lack of cerebral autoregulation (Ejlersen et al., 1994). However, similar reductions of ~30% relative to the pre-operative S_c_O_2_ were seen with IVC clamping in patients with and without encephalopathy (Panzera et al., 2006).

When the transplanted liver is reperfused, the brain can be subjected to hyperemia due to enhanced CO_2_ reactivity and/or liberation of vasodilating substances (Ejlersen et al., 1994) as we demonstrated by the 0.7 kPa increase in EtCO_2_ and if untreated can have adverse effects and affect even mortality (Skak et al., 1997). With impaired cerebral autoregulation, the risk of hyperperfusion is even larger due to missing cerebral vasoconstriction in response a 90% increase in CO and be aggravated by the vasodilatory effect of CO_2_ (Figure 1). Accordingly, S_c_O_2_ may guide to what extent VE should be increased in order to protect the brain. We observed an increase in S_c_O_2_ by 5.5% (3.8–7.3%) during reperfusion although VE was increased by 0.6 l/min. We, therefore, suggest a more meticulous control of VE is in need, as guided by EtCO_2_, until the end of LTx (Nissen et al., 2010). Although EtCO_2_ was kept within 4.6–6.0 kPa (Pott et al., 1995; Zheng et al., 2012) or VE increased by 15% (Pere et al., 2000), CBF becomes elevated (by more than 80% in some patients) with reperfusion of the liver, which emphasizes that attempts to maintain EtCO_2_ toward the end of the operation could attenuate cerebral hyperperfusion (Pott et al., 1995; Philips et al., 1998; Zheng et al., 2012).

The P_a_CO_2_ relates to hydrogen ion concentration and is a potent modulator of cerebrovascular resistance and, thus, CBF (Lassen, 1959). Hypercapnia leads to cerebral vasodilation while the opposite occurs with hypocapnia through a serial of endogenous mediators (Eriksson et al., 1983). In healthy humans, CBF increases 2–8% per mmHg CO_2_ as determined by Fick's principle (Kety and Schmidt, 1946) or transcranial Doppler (Madsen and Secher, 1999), however, CO_2_-reactivity has not yet been describe for NIRS despite S_c_O_2_ does follow CBF induced by hypercapnia and hypocapnia (Rasmussen et al., 2007). As evaluated by ^133^Xenon clearance in patients undergoing LTx, CBF increases by 25% and may be more than can be explained by the increase in P_a_CO_2_ (Larsen et al., 1999). Increasing P_a_CO_2_ may mitigate the CO_2_-reactivity because of near-maximal cerebral vasodilatation or may be attributable to other vasodilating substances interfering with the effect of CO_2_ on the cerebral vasculature (Philips et al., 1998).

As this was a retrospective study, we did not evaluate neurological outcome. In related studies, neurological complications range from mild seizures to hemorrhage and stroke after LTx (Adams et al., 1987; Stein et al., 1992; Madsen and Secher, 2000; Pere et al., 2000; Zheng et al., 2012) and cerebral hemorrhage and anoxic-ischemic lesions are common at brain autopsy after LTx (Ferreiro et al., 1992). However, the evidence for improved neurological outcome by maintaining S_c_O_2_ during LTx remains sparse, although improved outcome is seen in cardiac (Slater et al., 2009; Ono et al., 2013; Colak et al., 2014; Harilall et al., 2014), abdominal (Casati et al., 2005), and orthopedic surgery (Ballard et al., 2012). An observational cohort study is underway investigating the relationship between perioperative desaturation during hepatic surgery or LTx and adverse postoperative events and length of ICU stay, but optimization of S_c_O_2_ in the anhepatic and reperfusion phase is not included (clinicaltrials.gov: NCT01458262). Although the adequacy of cerebral autoregulation and oxygenation can be monitored in the operating room, impaired CBF regulation may persist into the early postoperative phase (Larsen et al., 1999), but no study describes the efficacy of maintaining cerebral monitoring in the ICU after LTx (Ejlersen et al., 1994; Van Mook et al., 2005).

From this retrospective study, we conclude that despite adjustments of VE in the anhepatic and reperfusion phases of LTx, S_c_O_2_ changes occur that have the potential to expose patients to cerebral ischemia and/or hyperemia. We suggest that a ventilatory strategy guided by EtCO_2_ would keep S_c_O_2_ more stable during LTx.

## Author contributions

All authors contributed equally to the design, data analysis and interpretation, drafting the manuscript and critical revision. All authors approved the final version before submission.

### Conflict of interest statement

The authors declare that the research was conducted in the absence of any commercial or financial relationships that could be construed as a potential conflict of interest.
